# Genomics and precision medicine and their impact on endocrinology

**DOI:** 10.1530/EC-23-0106

**Published:** 2023-06-08

**Authors:** Constantine A Stratakis

**Affiliations:** 1 Human Genetics & Precision Medicine, Institute for Molecular Biology & Biotechnology (IMBB), Foundation for Research & Technology Hellas (FORTH), Heraklion, Greece; 2 ELPEN, Inc. Research Institute, Athens, Greece; 3 European University of Cyprus School of Medicine, Nicosia, Cyprus; 4 Medical Genetics, Henry Dunant Hospital, Athens, Greece; 5 National Institute of Child Health and Human Development (NICHD), National Institutes of Health (NIH), Bethesda, Maryland, USA

This is my first note as an Advisory Editor of our journal *Endocrine Connections* which seeks to expand coverage on the generic area of endocrine genetics. I am honored to be selected to assist in this role. I have served endocrinology and genomic medicine for more than three decades and I am delighted to see the developments of the latter and their impact on the former. *Endocrine Connections* is right to want to cover more endocrine genetics, as precision medicine (that is largely based on genomics) becomes the cornerstone of prevention, diagnosis, and treatment of almost all conditions affecting human metabolism and is part of everyday clinical practice in endocrinology.

Many of today’s leaders in Endocrinology, including myself, were trained in the 1980s and early 1990s when two advances revolutionized medicine: the first was theoretical; it was the introduction of the concept of ‘positional cloning’, the idea that one can identify genes for human disease without knowing anything (or with knowing very little) about their function. The second was technical; the method of polymerase chain reaction (PCR) made DNA (the genome in essence) available to biomedical researchers and, more importantly, clinicians. Cancer medicine and traditional human genetics were the fields that benefited most from the first applications of the new genomic concepts and technologies. The human genome project (HGP) led to the completion of the first genomic maps using mostly PCR-based Sanger sequencing. The latter was expensive, laborious, and impractical for studying whole genomes. As the 1990s came to a close, few in Endocrinology other than those studying rare diseases would have predicted what is going on today. Indeed, HGP technologies that grew out of necessity led to the development of next generation sequencing methods that are now widely available (1). Many more rare diseases were elucidated at the molecular level and the identification of single-nucleotide polymorphisms (SNP) led to genome-wide association studies that have revealed loci for the determination of endocrine traits such as height, menarche and menopause, and predisposition factors for diabetes and other endocrine diseases, to name just a few. SNP-based algorithms are now used for the calculation of polygenic risk scores (PRS) for a number of traits and diseases.

Thus, today, the combination of the knowledge of genetic causes of various forms of syndromes affecting the pituitary, thyroid, parathyroid, pancreas, adrenal, the gonads, and so on, and of genomic loci harboring risk alleles for common traits and a number of endocrine conditions makes the application of precision medicine in everyday clinical practice imperative (2). Like in other fields of medicine, the various omics are poised to alter the way we prevent, diagnose, and treat endocrine conditions (3). We now have the opportunity not only to understand cellular processes, glandular development, and disease pathophysiology but also to apply molecularly designed treatments (4). The changes are fast and present us with new challenges from the protection of personal data to the interpretation, implementation, and overall use of genomic information (5).

The continuous shifting of ideas and practices is indeed very real in modern medicine and endocrinology and is due to the advances in genetics. There are no better examples of this than the far-reaching effects of discoveries from the UK genomic data (6), discoveries that are now multiplying from similar efforts in other settings, such as the most recent studies of the Finnish population (7). Hippocrates noted that “Medicine cannot be learned quickly because it is impossible to create any established principle in it, the way that a person who learns writing according to one system that people teach understands everything; for all who understand writing in the same way, do so because the same symbol does not sometimes become opposite, but is always steadfastly the same and not subject to chance. Medicine, on the other hand, does not do the same thing at this moment and the next, and it does opposite things to the same person, and at those things that are self-contradictory” (8).

*Endocrine Connections* is poised to lead in studies from this new world in precision medicine and its applications in endocrinology; I and my colleagues on the Editorial Board of the journal are humbled by the task and honored to be involved in the process.

## Declaration of interest

The author reports funding from the ELPEN, Pfizer, Lundbeck, and Sterotherapeutics pharmaceuticals, Human Longevity, Inc., and holds patents on the PRKAR1A, PDE11A and GPR101 genes and functions.

## Funding

Dr. Stratakis is funded through IMBB, FORTH, Heraklion, Greece, and in part by intramural NICHD, NIH, Bethesda, MD, USA.
